# Urban thermal data analysis over the period 1948–2022: a case study of Ljubljana, Slovenia

**DOI:** 10.1007/s00484-025-03021-3

**Published:** 2025-09-12

**Authors:** Zalika Črepinšek, Zala Žnidaršič, Tjaša Pogačar

**Affiliations:** Biotechnical Faculty, University of Ljubljana, Jamnikarjeva 101, Ljubljana, 1000 Slovenia

**Keywords:** Extreme high temperatures, Climate indices, Heat sum, Heat waves, Urban heat exposure, Ljubljana

## Abstract

The aim of the study was to characterize the intensity, frequency and duration of extreme high temperature events and their variability over a period of 75 years (1948–2022) for Ljubljana, Slovenia. This study uses 23 thermal indices recommended by the WMO (ETCCDI) based on daily maximum and minimum air temperatures, retrieved from the Slovenian Environment Agency. The study conducted showed an increase in heat stress risk during the summer months over the last 75 years, with particularly pronounced changes since the 1990s. The observed increase in air temperature was greater for extreme than for average temperatures. The trends in annual average maximum, minimum and daily temperatures were all positive and significant with rates of 0.37 °C/decade, 0.41 °C/decade and 0.39 °C/decade respectively. As a result of these changes, the number of hot days, tropical nights, intensity, frequency and duration of heatwaves (HW) have also increased. HW are becoming a growing problem in Ljubljana, as all HW indices examined are increasing: number of HW (trend 0.5 events/decade), frequency (2.0 days/decade), magnitude (0.36 °C/decade) and maximum amplitude (0.73 °C/decade). Until recently, these events were only typical of summer, but now they occur in May and even last into September. The thermal heat sum indices, heating degree days (HDDheat) and cooling degree days (CDDcool), indicators of weather-related energy consumption for heating and cooling buildings, showed a clear change, namely a decrease in HDDheat and an increase in CDDcool. The city has experienced pronounced urban growth, which is accompanied by significant changes in the area surrounding the measurement site, which, together with climate change, exacerbate the risk of heat exposure. Despite numerous measures already taken to reduce heat stress in the city, it remains a problem in the summer months, especially given the prediction that conditions will worsen in the future. It is therefore necessary to continue monitoring temperature conditions and local and temporal changes, which is the responsibility of the National Meteorological Service. Further studies on urban characteristics and human thermal comfort parameters are also needed to assess local vulnerability. In addition, some complementary measurements could be carried out to collect data on spatial variations, which is an important step in developing a plan to combat heat stress.

## Introduction

The proportion of the world’s population living in cities has doubled from 25% in 1950 to 56% in 2021 and is projected to rise to 68% by 2050 (Martins and Sharifi 2022). The share of the urban population in Europe is much higher than the global average and even amounted to three quarters of the total population in 2021 (The World Bank 2023). According to the United Nations World Cities Report 2022, all regions of the world are expected to become more urbanized in the coming decades. The European urban population is expected to grow by 0.26% annually between 2015 and 2055. The report also states that climate change is the greatest threat and risk to urban health, resulting in more intense, frequent and prolonged extreme weather events. Air temperatures in Europe have risen significantly over the period 1991–2021, by an average of around 0.5 °C per decade, which is the largest change of any WMO region (The State of the Climate 2023). The number of studies on climate change and its impact on urban climate has increased in recent decades, as cities are particularly vulnerable to weather-related extremes due to their densely populated city centers, concentrated buildings and infrastructure. The study on the impact in European cities found that mortality during heat waves increased from 8 to 34%, with large geographical differences between cities (D’Ippoliti et al. 2010). Hot extremes, including heatwaves, have increased in cities, where they have impacts on human health and well-being, transportation, water supply, sanitation, energy systems and air quality (IPCC 2022).

The WMO Regional Climate Centre for Europe recently published an overview of heatwaves since 1950, showing that of the 23 most severe heatwaves, 16 occurred after the year 2000 (Martins and Sharifi 2022). The likelihood of extremely hot summers has increased dramatically since the European heatwave of 2003 (Christidis et al. 2015). A comprehensive study by Smid et al. (2019) has shown that the frequency and severity of extreme heat events increased significantly in all European capitals between 1981 and 2010. In addition, Europe has been identified as a heatwave hotspot, showing an upward trend three to four times faster than the rest of the northern mid-latitudes over the last four decades (Rousi et al. 2022).

Urban surfaces generally have a large heat storage capacity, which largely explains the urban heat island (UHI) effect, the phenomenon whereby temperatures in densely built-up cities are higher than in neighbouring suburban rural areas (Varquez and Kanda 2018). Overheating poses a multifaceted threat to the health and well-being of urban populations as well as to energy efficiency and thus the cost of living in cities (Nazarian et al. 2022). Urban overheating has recently been documented for more than 400 major cities around the world. The experimental data collected showed that the magnitude of the average temperature increase can exceed 4–5 °C and peak at 10 °C (Santamouris 2020). Scientists from the European Commission’s Joint Research Centre studied the urban heat island effect for cities with more than 50,000 inhabitants in the summers between 2003 and 2020 and found that surface temperatures in cities were sometimes even up to 10–15 °C higher than in their rural surroundings (Mentaschi et al. 2022). Numerous studies have confirmed that the UHI effect has intensified in recent decades, with a stronger effect during nights and at higher latitudes (IPCC 2022; Mentaschi et al. 2022; Smidt et al. 2019; Varquez and Kanda 2018). Projections indicate that European cities will be more vulnerable to extreme heat in the coming decades, but even more so in the distant future. Extreme heat events in European capitals will not be limited to the regions already most affected today, such as the Mediterranean or the Iberian Peninsula (D’Ippoliti et al. 2010; Smid et al. 2019), the largest temperature increases during heatwaves are expected in Central European cities (Guerreiro et al. 2018).

Although Ljubljana’s population is relatively small compared to other European capitals (approx. 300,000 inhabitants in 2023) (SURS 2024), the city faces problems in summer due to the urban heat island. Temperatures in Ljubljana above 35 °C are becoming more frequent, the highest temperature was recorded in the summer of 2013 when it even exceeded 40 degrees, and the annual number of hot days (Tmax ≥ 30 °C) may exceed value 50 (ARSO 2024b).

The parameters of the summer average daily temperature distributions for the city have changed, the mean values have shifted from 19.1 °C (1961–1990) to 21.1 °C (1991–2020) (ARSO 2024b). Studies of temperature conditions have shown that Ljubljana has a pronounced urban heat island in the order of over 5 °C, with some of its districts consistently warmer than others, which are defined as hotspots (Komac et al. 2016). In recent decades, urban development, especially the construction of many shopping centers with large parking lots, has increased the intensity of the urban heat island. Heatwaves have been a growing problem recently; their number and intensity increased after 1990. Around 20 to 30 days of heatwaves every summer have become a constant in Ljubljana (ARSO 2024a; Pogačar et al. 2018). The period in which heatwaves occur has also lengthened, because until the 1990s they did not occur at the beginning of June and end of August as they did recently (ARSO 2024b). The percentage of summer days in the period 2000–2021 with severe or very severe heat stress for Ljubljana is around 30% (Črepinšek et al. 2023) and analyses of various bioclimatic indicators (wet bulb temperature, Universal Thermal Climate Index, Water Loss Index, Assumed Level of Physical Activity) confirmed that on most days of heatwaves there is a high risk of heat stress with moderate or severe stress (Črepinšek et al. 2023; Pogačar et al. 2020). Heatwave-related deaths have increased in recent years; the most vulnerable population during heatwaves in Slovenia is the elderly (Perčič et al. 2018) and in the largest urban areas, the cities of Ljubljana and Maribor (Žiberna et al. 2021), mortality during heatwaves is higher than in rural areas (Grašič and Perčič 2022). Heat-related mortality is a major concern, but it is also necessary to monitor the burden on the healthcare system in terms of morbidity indicators such as emergency department visits, hospitalisations or ambulance transports (Dwyer et al. 2022; Perčič et al. 2024). The studies should include different types of activities, such as predicting morbidity before a heat wave, timely monitoring during a heat wave and assessing health effects after a heat event (European Climate 2024).

Unfortunately, the climate projections are also not promising in terms of extreme temperatures for Ljubljana (ARSO 2019). The results of the climate models were used to assess the change in the number of heatwave days and the change in the maximum temperature of heatwaves (ΔTmax) between the historical (1951–2000) and future (2051–2100) period for 571 European cities from the “GISCO Urban Audit 2004” dataset (Guerreiro et al. 2018). In this study, Ljubljana is expected to experience a 15.3% change of heatwave days and a ΔTmax of + 6.4 °C according to the low impact scenario, and 47.6% more heatwave days with a ΔTmax of + 12.3 °C according to the high impact scenario. As the above research has not considered possible changes in urbanisation and thus the urban heat island, this could mean even greater changes in the future.

Smid et al. (2019) calculated heatwave indices with the EURO-CORDEX ensemble for historical (1971–2005) and future (2006–2100) periods under the RCP8.5 scenario for all European capitals. In the near future (2021–2050), the occurrence of heatwaves with a magnitude > 6 (“extreme” events) is also more likely in Ljubljana, and in the distant future (2071–2100), the probability of occurrence of heatwaves with a magnitude of more than 9 (“very severe” events) is > 0.75. According to the mentioned study, Ljubljana will experience a dramatic increase in heatwaves.

Air temperatures in Ljubljana have risen in recent years, and the number and intensity of heatwaves are increasing (ARSO 2024b; Vertačnik et al. 2018), but Slovenia still does not have a comprehensive heat warning system that combines public health measures with meteorological forecasts (Pogačar et al. 2020). Due to the high risk to human health and the negative impact on well-being and productivity at high temperatures in this urban area, additional research is needed to better understand thermal conditions in cities. The aim of this study was to characterize the intensity, frequency and duration of extreme temperature events and their variability over a 75-year period (1948–2022) for Ljubljana. This study uses 23 indices recommended by the WMO Expert Team on Climate Change Detection and Indices (ETCCDI) calculated with the Climpact-v2 software. For each index, we calculated the type of trend and significance using the Mann–Kendall test and the magnitude of the trend using Sen’s slope estimator. This methodology and the results will support local authorities in decision-making regarding the development strategy in the city and serve as a basis for the national climate change mitigation and adaptation strategies. The use of a standardised set of indicators and a standardised methodology offers the possibility of international comparison. Furthermore, the inclusion of thermal sum indices directly related to building energy consumption of buildings adds an important dimension to the study in terms of urban planning and thermal comfort, energy policy and the development of strategies to reduce vulnerability to climate change. The results of our study at the local level can serve as validation and calibration data for large-scale modelling and can be further explored in future studies and compared with data from other studies to enable more detailed temporal and local analyses.

## Materials and methods

The Expert Team on Sector specific Climate Indices (ET-SCI) and the Expert Team on Climate Change Detection Monitoring and Indices (ETCCDMI) under the direction of the World Meteorological Organization (WMO) have developed indices for the investigation of thermal conditions. We used Climpact-v2 software written in R, a language and environment for statistical computing and graphics, to calculate 23 thermal indices. The definitions of these indices are listed in Table 1 (Alexander and Herold 2016). Of all the indices available in the application, we used only those relevant to the assessment of thermal conditions at high air temperatures.

**Table 1:** Calculated temperature indices with definitions

Indices (Code)	Name	Definition
TMm	Mean TM	Mean daily mean temperature (°C)
TNm	Mean TN	Mean daily minimum temperature (°C)
TXm	Mean TX	Mean daily maximum temperature (°C)
TNn	Min TN	Annual minima value of daily TN (°C)
TNx	Max TN	Annual maxima value of daily TN (°C)
TXn	Min TX	Annual minima value of daily TX (°C)
TXx	Max TX	Annual maxima value of daily TX (°C)
DTR	Daily temperature range	Annual Mean difference between daily TX and TN (°C)
SU25	Summer days	Annual number of days when TX > 25 °C
SU30	Hot days	Annual number of days when TX ≥ 30 °C
SU35	Very hot days	Annual number of days when TX ≥ 35 °C
TR	Tropical nights	Annual number of days when TN ≥ 20 °C
WSDI	Warm spell duration indicator	Annual number of days with at least 6 consecutive days when TX > 90th percentile
TX90p	Amount of hot days	Percentage of days when TX > 90th percentile
TN90p	Amount of hot nights	Percentage of days when TN > 90th percentile
HDDheat	Heating degree Days	Annual sum of Tb - TM (Tb is a user-defined location-specific base temperature of 12 °C and TM < Tb) (°D; degree-days)
CDDcool	Cooling degree Days	Annual sum of TM - Tb (Tb is a user-defined location-specific base temperature of 21 °C and TM > Tb) (°D; degree-days)
GDDgrow	Growing degree Days	Annual sum of TM - Tb (Tb is a user-defined location-specific base temperature of 5 °C and TM > Tb) (°D; degree-days)
HWN (Tx90p)	Heatwave number (HWN), defined by 90th percentile of TX	The number of individual heatwaves that occur each from May to Sep, defined as 3 or more days where TX > 90th percentile of TX
HWF (Tx90p)	Heatwave frequency (HWF), defined by 90th percentile of TX	The number of days that contribute to heatwaves as identified by HWN
HWD (Tx90p)	Heatwave duration (HWD), defined by 90th percentile of TX	The length of the longest heatwave identified by HWN (days)
HWM (Tx90p)	Heatwave magnitude (HWM), defined by 90th percentile of TX	The mean temperature of all heatwaves identified by HWN (°C)
HWA (Tx90p)	Heatwave amplitude (HWA), defined by 90th percentile of TX	The peak daily value in the hottest heatwave (defined as the heatwave with highest HWM) (°C)

The data used in the Climpact-v2 software include the daily minimum and maximum temperatures for the period 1948–2022 at the meteorological station Ljubljana (46.0655°N, 14.5124°E, 299 m), which were provided by the Slovenian Environment Agency ARSO (ARSO 2024b). The climate of Ljubljana is continental, “Cfb” according to the Köppen climate classification, with warm summers and moderately cold winters. In the period 1981–2010, the average annual temperature was 10.9 °C and the amount of precipitation was 1362 mm. July and August are the warmest months with daily highs between 25 and 30 °C. The highest temperature recorded in Ljubljana is 40.2 °C, measured on August 8, 2013. Due to its location in a basin, natural urban ventilation in Ljubljana is often limited during temperature inversions. The average wind speed is low; the long-term average for the summer months is 1.4 m/s.

For three indices (Heating, Cooling and Growing degree indices, called HDDheat, CDDcool, GDDgrow) there is an option for a user-defined site-specific base temperature. Following the thresholds used by the ARSO in its applied research for Slovenia (ARSO 2024c), we have chosen a base temperature (Tb) of 12 °C for the HDDheat index, 21 °C for the CDDcool index and 5 °C for the GDDgrow index. HDDheat and CDDcool indices are used to predict the heating and cooling requirements of residential buildings where it is important to accurately analyse meteorological or climatic parameters when designing indoor thermal comfort. They are indicators of locations that are thermally comfortable and have low heating and cooling demand and also serve as indicators of predicted climate change (Petri and Caldeira 2015). In addition, some researchers have suggested using heating and cooling degree days as indicators of the characteristics of the urban heat island effect (Tian et al. 2021). Growing degree days (GDD), which measure heat accumulation during the growing season, are an important indicator of plant and pest development, which is related to many aspects of human health, such as the consequences of longer pollen seasons that cause allergies, or GDD models are used, for example, to estimate the number of mosquito generations, which is closely related to mosquito-borne diseases (Mushegian et al. 2021).

The quality control of the data was already carried out as a regular procedure when archiving the data at ARSO, but a quality control and homogeneity check of the daily input data was performed again with the Climpact-v2 software before calculating the climate indices to check for possible input errors or missing data. A trend analysis was also performed for all indices using the non-parametric Mann-Kendall (MK) test and Sen’s Kendal Slope Estimator (Rahman and Dawood 2017; Sen 1968), with statistical significance at the 5% level over 75 years (1948–2022). MK is widely used in climatological studies, it is simple and robust, has no data distribution requirements and can handle missing values (Salmi et al. 2002). The two-tailed MK test with a significance level of α = 5% was applied in our study to assess the significance of the temperature trends. After evaluating the statistical significance of the trends, we accurately determined the magnitude of the trends in the climate indices using Sen’s test. Sen’s slope reflects the median slope and is more suitable for calculating trends at extremes than other common trend estimators because it is less influenced by outliers (Aleksander and Herold 2016). In addition to the slope, we also calculated the confidence interval (lower and upper bound) at 1–α = 95% probability level. The MK test was performed using XLSTAT from Addinsoft (2023) and the Sen test using the software Climpact-v2, available for free at https://climpact-sci.org/.

## Results

### Mean temperatures and diurnal temperature range

The results of our analysis are summarized in Table 2. The trends in annual mean maximum (TXm), minimum (TNm) and mean daily temperatures (TMm) were all positive and statistically significant with a Mann-Kendall p-value < 0.0001. All magnitudes of the trends are given with Sen’s slope estimators in Table 2. The increase in TMm over the last 75 years was almost 3 °C–0.39 °C per decade. The changes in TXm and TNm show a very similar pattern with a warming trend of 0.37 °C and 0.41 °C per decade, respectively. Given the similar increase in TXm and TNm, the trend in the diurnal temperature range (DTR) is statistically insignificant (*p* = 0.131), which means that the average difference between the daily maximum and minimum temperatures has not changed noticeably over the period studied.

**Table 2:** Calculated annual temperature indices with results of the Mann-Kendall (MK) and sen’s slope estimator tests for Ljubljana in the period 1948–2022

Indices (Code)	Mean (SD)	Max	Min	Sen’s slope	Lower; upper limits	MK-test *p*-value	MK-test trend
TMm	10.8 (1.0)	13.2	8.9	0.039	0.031; 0.047	< 0.0001	***
TNm	6.2 (1.0)	8.6	4.3	0.041	0.035; 0.048	< 0.0001	***
TXm	15.5 (1.1)	18.3	13.6	0.037	0.026; 0.046	< 0.0001	***
TNn	−13.0 (3.7)	−6.0	−23.3	0.093	0.061; 0.125	< 0.0001	***
TNx	19.7 (1.4)	23.8	16.4	0.050	0.039; 0.059	< 0.0001	***
TXn	−5.3 (2.5)	0.0	−12.1	0.041	0.016; 0.067	0.002	+
TXx	34.1 (2.0)	40.2	29.6	0.048	0.029; 0.065	< 0.0001	***
DTR	9.3 (0.6)	10.3	7.8	−0.005	−0.012; 0.001	0.131	n.s.
SU25	68 (16)	113	36	0.484	0.333; 0.600	< 0.0001	***
SU30	18 (12)	54	0	0.344	0.233; 0.450	< 0.0001	***
SU35	1.2 (2.4)	11	0	0.000	0.000; 0.000	< 0.0001	***
TR	1.5 (2.8)	14	0	0.029	0.000; 0.045	< 0.0001	***
WSDI	7.1 (9.2)	37	0	0.048	0.000; 0.206	< 0.0001	***
TX90p	9.9 (5.8)	26.9	1.4	0.167	0.113; 0.218	< 0.0001	***
TN90p	9.8 (6.2)	28.0	0.8	0.228	0.183; 0.271	< 0.0001	***
HDDheat	1493 (226)	2011	879	−7.044	−9.070; −4.985	< 0.0001	***
CDDcool	87.1 (64)	271.5	8.1	2.076	1.634; 2.518	< 0.0001	***
GDDgrow	2601 (268)	3219	2126	9.954	8.173; 11.828	< 0.0001	***
HWN (Tx90p)	2.0 (2.1)	11	0	0.044	0.023; 0.062	< 0.0001	***
HWF (Tx90p)	9.2 (9.9)	53	0	0.203	0.117; 0.275	< 0.0001	***
HWD (Tx90p)	5.6 (2.0)	13	2	0.024	0.000; 0.048	0.027	+
HWM (Tx90p)	30.8 (2.3)	35.0	25.7	0.036	0.007; 0.065	0.037	+
HWA (Tx90p)	33.5 (3.0)	40.2	27.0	0.073	0.032; 0.105	0.001	**

The upper and lower confidence limits for Sen’s slope at 1-α = 95%, MK-Mann-Kendall trend test, n.s. non-significant, +significant at 5%, **significant at 0.1%, ***significant at 0.01%

### Indices of extreme temperature intensity

**Fig. 1:**
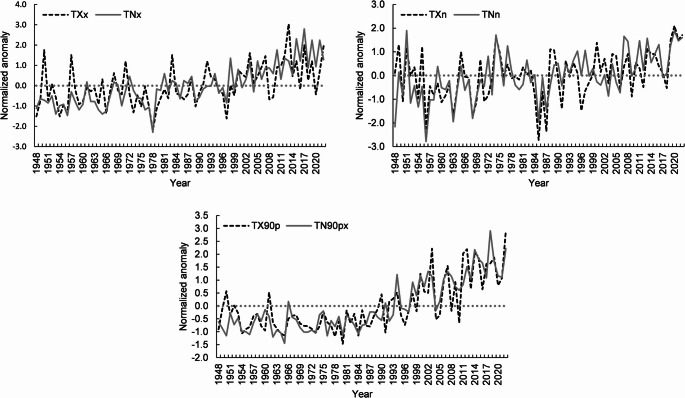
Change in the anomaly series of the extreme temperature indices: maximum value of the daily maximum temperature (TXx), minimum value of the daily maximum temperature (TXn), maximum value of the daily minimum temperature (TNx) and minimum value of the daily minimum temperature (TNn), percentage of days on which TX > 90th percentile (TX90p) and percentage of days on which TN > 90th percentile (TN90p). The descriptions of the indices and the results of the Mann-Kendall test trend can be found in Tables 1 and 2

‘In the 75-year period under consideration from May to September, when the probability of the greatest heat is highest, the lowest absolute temperature of −2.8°C was recorded in Ljubljana in May 1956 and the highest in August 2013 at 40.2°C. Both the annual minimum value of the daily TN (TNn) and the annual maximum value of the daily TN (TNx) have increased significantly in recent years, which shows that the absolute coldest and warmest nights of the year in Ljubljana are warmer today (Table 2, Fig. 1). According to the calculated Sen’s slopes of 0.093 and 0.050, this means an increase that reaches a maximum of almost 7 °C for the coldest nights and a smaller increase of 3.8°C for the warmest nights during the study period. The number of hot nights (TN90p) has also increased. The percentage of days on which the TN value is above the 90th percentile has recently been significantly higher, with an increase rate of 2.3% per decade (*p* < 0.0001). On average, there are around 10% “unusually hot” nights per year, i.e. they are in the upper 10th percentile. Both the lowest maximum temperatures of the year (TXn) and the highest maximum temperatures (TXx) are also increasing. However, these extreme temperature changes during the day are lower (3.1°C and 3.6°C) than at night. The average annual percentage of hot days (TX90p) is the same (10%) as that of hot nights, but the change is slightly smaller with an increase rate of 1.7% per decade (*p* = 0.0001).

### Events with a fixed threshold temperature

Events with a fixed threshold for the maximum temperature, summer days (SU25, TX > 25 °C), hot days (SU30, TX ≥ 30 °C) and very hot days (SU35, TX ≥ 35 °C) showed statistically significant positive trends. Ljubljana has an average of 68 summer days per year in the 75-year period under consideration, but in the period 2013–2022 this average was significantly higher at 88 days. The Sen’s slope is 0.48, which means an upward trend of 36 days in the 75-year period under consideration. Prolonged exposure to temperatures above 25 °C combined with high humidity can already lead to heat stress in humans (Asseng et al. 2021). Once the temperature exceeds a person’s tolerance range, which is generally considered to be > 30 °C, it begins to affect the body’s performance (Chen and Qin 2022). In Ljubljana, there is an average of 18 days per year with TX above 30 °C, during the study period the annual number of SU30 increased by 26 days. The two most extreme years in terms of hot days were 2003, when there were 54 such days, with 23 in just one month (August), and 2022 with 51 hot days, in 2024, which was not included in the survey, there were 53 such days (ARSO 2024b).

The results of the analysis also show that hot days, which for a long time were typical only for the summer months, have been occurring as early as May in recent years and even persist in September. The duration of the period of high heat stress is getting longer, and there are also more frequent periods of several consecutive days when the TX does not fall below 30 °C. In the summer of 2013, for example, there were 19 consecutive hot days in Ljubljana from July 22 to August 9. Climate projections for Slovenia show with great reliability (ARSO 2019) that hot days will also occur more frequently in spring and fall. Unusually high temperatures in October already occur in some regions of Slovenia, e.g. a record temperature of 31.3 °C was measured in Črnomelj with a continental climate in October 2023; Ljubljana also reached an unusually high temperature of 27 °C for this period in the same week (ARSO 2024b). On 7 April 2024, 30.2 °C was measured in Osilnica with a continental climate, which is also the earliest 30 °C measured in a calendar year in Slovenia; on the same day, the highest temperature in Ljubljana was 29 °C (ARSO 2024b). Until the year 2000, days with TX above 35 °C were very rare in Ljubljana. In the first 50 years of the period under consideration, there were only 6 years in total with very hot days. After 2000, such days became a constant, and from then until 2022 there were 16 years with at least one SU35. In the period 1948–2012, we could expect a very hot day only once every three years, and the average for the period of the last 10 years, 2013–2022, is 4 very hot days per year. The largest annual number of days with TX above 35 °C occurred in the summer of 2013, namely 11. In August 2013, the absolute highest air temperature for the entire measurement period since 1851 was measured in Ljubljana, namely 40.2 °C (ARSO 2024b).

Of the 75 data points in our time series, the majority of years have no TX values above 35 °C, only 22 have non-zero values. The large number of zero values in the time series explains the difference between Sen’s slope (0.00) and the MK test trend, which is significant at 0.01% (Table 2). In an urban environment, buildings often overheat during heatwaves during the day, and at night the atmosphere cools less than in nearby rural areas due to heat emission. The number of tropical nights (TR) in which the lowest daytime temperature does not fall below 20 °C has increased significantly at a rate of 0.3 days per decade (*p* < 0.0001), with a sharp increase since 2000. In the period under consideration, there were only 5 years in the first 45 years in which 1 tropical night was recorded, but in the last 10 years the annual average rose to 7 tropical nights. We had the most TRs in the summer of 2015, namely 14 days, 12 of which were in the month of July. July is also the month with the most tropical nights on average, almost half of all such events (48%), followed by August with 34% and June with 18%. Until 2002, we did not record any tropical nights in June, but in the last 20 years this has become a normal occurrence, with at least one tropical night on average in June. Therefore, both the annual number of TRs and the length of the period in which TRs can be expected are increasing.

### Cumulative temperature sums

**Fig. 2:**
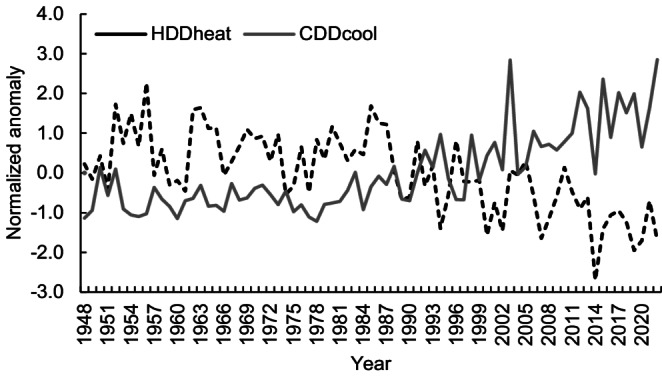
Change in the anomaly series of the cumulative temperature sum indices: Heating Degree Days (HDDheat) and Cooling Degree Days (CDDcool) with pronounced changes since the late 1980s. The descriptions of the indices and the results of the Mann-Kendall test trend can be found in Tables 1 and 2

The indices of Heating degree days (HDDheat) and cooling degree days (CDDcool), which contribute to the interpretation of energy consumption for heating and cooling buildings, have changed significantly in Ljubljana in recent times (Fig. 2). The mean value of HDDheat data from 75 years is 1493 °D, with a considerable change between the period until 1990 with HDDheat 1620 °D and the period 1991–2022, when these values are much lower, on average 1324 °D. The trend shows that the heating degree days decrease significantly over time. The HDDheat values fell by 18% between the first and second period. A particularly pronounced change is characteristic after 1987. After that, the HDDheat values were lower than the average in all but five years (2010, 2005, 1996, 1993 and 1991). Interannual variability is also very high. In the coldest period of 1956, the annual GDDheat was 229% of that of 2014.

In contrast, the CDDcool values increase over time. For the period 1991–2022, these values (143 °D) were more than three times higher than for the period 1948–1990 (46°D), indicating that cooling demand in buildings has increased in recent decades. In recent years, the CDDcool values are already approaching the value of 300 °D, e.g. the CDD in 2003 was 271 °D and in 2022 the absolute extreme of 272 °D was reached.

GDDgrow, a measure of the accumulated heat energy that plants and pests need to grow and develop, a widely used measure of the intensity of the thermal growing season, showed a significant positive trend, especially pronounced after 1990, very similar to HDDheat and CDDcool. The average rate of change in Ljubljana was an additional 10 °D per year, which means that the original sum of GDD has increased from 2225 °D to almost 3000 °D in recent years.

### Heatwave indices

**Fig. 3:**
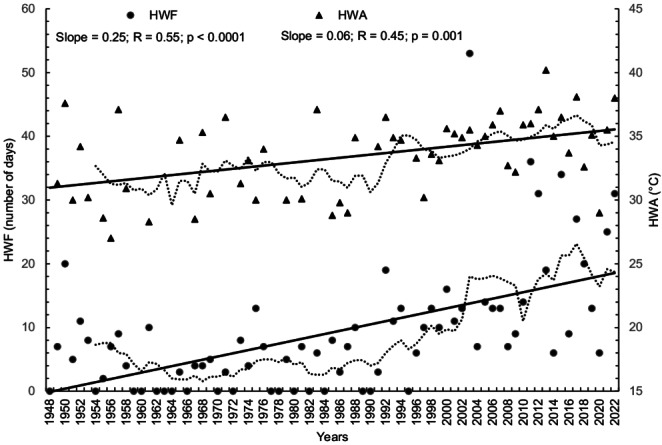
Long-term trends in the frequency of heatwaves in days (HWF) and the amplitude of heatwaves in °C (HWA). The descriptions of the indices and the results of the Mann-Kendall test trend can be found in Tables 1 and 2. Straight lines represent statistically significant linear regressions and the dashed line is the smoothed 7-year running average

The total number of heatwaves (HWN) for the first analyzed quarter-century period P1 (1948–1972) was 0.9 events/year and 4.0 events/year for the third analyzed quarter-century period P3 (1998–2022), with an average significant increase rate of 0.5 events/decade. The change is even greater when looking at the HWF (heat wave frequency) for Ljubljana, with a fourfold increase in HWF days in the P3 period compared to the first P1 period, on average from 4.1 to 18.0 days/year (Table 3; Fig. 3), with the HWF increasing at a rate of 2.5 days/decade. The length of the longest HW (HWD) in the first two periods in Ljubljana was 8 days, in P3 the longest heat wave in 2003 lasted a total of 13 days (Table 3). In recent years, the average heatwave in Ljubljana has been more than six days long, which is more than one day longer than the average heatwave in the 1950s. Another important fact is that after 1995 there was no year without a heatwave, which was quite common in the first years analyzed. The mean annual HW magnitude (HWM) and the maximum HW amplitude (HWA) have also increased significantly, with positive rates of change for HWM 0.36 °C/decade and for HWA 0.73 °C/decade. The mean temperature of all heatwaves in P3 is 1.3 °C higher than in P1, and the increase in the daily peak in the hottest heatwave is even more extreme. TX in the hottest heatwave in P1 did not exceed 36.5 °C, but in the last 10 years these values are significantly higher. The highest value in the entire period under consideration was reached during the heatwave in August 2013 (40.2 °C), and the second and third highest HWA values were reached in 2017 (38.1 °C) and 2022 (38.0 °C). A warm spell duration, i.e. a period of at least six consecutive days on which the daily TX value exceeds the 90th percentile, lasted on average one week over the entire period under consideration. A significant positive trend was observed for WSDI (+ 3.6 days), although the values increased mainly after 2003 and amounted to 17 days for this period. The index shows that the high air temperatures in Ljubljana have persisted for several weeks in recent years, with the highest values recorded in 2014, 2015 and 2022.

**Table 3:** Characteristics of heat wave (HW) indices for periods of 25 years: total number of HW (HWN), total duration of HW in days (HWF), length of the longest HW in days (HWD), mean temperature of all HW in °C (HWM), maximum temperature in the hottest HW in °C (HWA) and total duration of events with 6 or more consecutive days of TX90p in days (WSDI)

	HWN	HWF	HWD	HWM	HWA	WSDI
P1 (1948–1972)	22	102	8	30.1	36.5	88
P2 (1973–1997)	31	140	8	30.5	37.1	72
P3 (1998–2022)	100	450	13	31.4	40.2	369

## Discussion

This study showed an increase in summer heat exposure in Ljubljana over the last 75 years, which is consistent with other global studies (Christidis et al. 2015; Klein and Anderegg 2021; Tuholske et al. 2021) as well as with previous studies at the local level (Črepinšek et al. 2023; Grašič and Perčič 2022; Komac et al. 2016). The observed increase in air temperature was greater for the extreme temperatures than for the average temperatures. Due to these changes, the number of hot days, tropical nights, intensity, frequency and duration of heatwaves have also increased. Today, the inhabitants of Ljubljana face air temperatures of more than 35 °C in summer, which was an extremely rare phenomenon two decades ago (ARSO 2024b). Heatwaves start earlier and can last until late fall – citizens are exposed to the heat for longer, which worsens the quality of life in the city. In the last 20 years, the city has had 14 of the warmest summers in the three-quarter century period in question, and the problem of heating the city has become more pressing, which is also true for other European capitals (Smid et al. 2019). In a recent study in the region of Augsburg, Germany, the analysis shows that extreme heat at night increases the risk of stroke by 7% (He et al. 2024). This is important because climate change is causing nighttime temperatures to rise much faster than daytime temperatures (Zhong et al. 2023). Given the recent increase in tropical nights in Ljubljana, additional measures will be needed to protect citizens from the effects of night-time heat.

As for the cumulative temperature sums analyzed, the changes observed for Ljubljana are consistent with the results for Europe, where the heating demand for a given building in 2022 was about two tenths lower than in 1979 and the trend shows that heating degree days are decreasing significantly over time (Eurostat 2024). Cooling degree days have increased significantly worldwide (Scoccimarro et al. 2023). In the EU Member States, the trends for CDD observed in the measurements show an increase over time. According to Eurostat (2024), CDD values in 2022 were almost four times higher than in 1979, indicating an increased need for building cooling. Due to global warming, a further increase in energy demand for cooling in summer is predicted, while the demand for heating in winter is expected to decrease (Karagiannids et al. 2023; Spinoni et al. 2018). Although the projections for Slovenia clearly show that the energy demand for cooling will increase in the warmer seasons and the demand for heating will decrease in the colder seasons due to climate change (Climate Change 2024), some other influencing factors must also be considered. For example, a case study for the heating demand in Slovenia adjusted for population aging has shown that the gap between the reference scenario and the age-adjusted scenario widens over time as the proportion of older people increases (Lampret et al. 2019). Other adaptation measures should also be included, many of which are already covered by the main objectives of the Ljubljana Sustainable Urban Strategy of Ljubljana, such as the energy renovation of public buildings owned by the City of Ljubljana, including thermal insulation of facades, replacement of building joinery, ceiling insulation of unheated attics, introduction of modern energy management, sustainable construction in the future and restoration of degraded land (Sustainable Urban Strategy 2020). In recent years, increased attention has also been paid to improving education about heat risks (Pogačar et al. 2020) and alerts (ARSO 2024a) with new communication technologies for all citizens, training of workers and raising public awareness of heat stress (Hojs et al. 2024; Hotspots 2024).

Many studies have confirmed that thermal resources have recently increased and that this has an impact on the growing season (Wypych et al. 2017). Spinoni et al. (2015) examined the development of GDD in Europe over the period 1951–2011 and found that there were significant positive trends in most parts of Europe, with the largest changes over the last 30 years in the Mediterranean region and the smallest in Northern Europe. In the above study, the thermal energy available for plant and animal growth increased by 6.2 °D year^−1^ in Italy, 5.2 °D year^−1^ in the Alps and 4.4 °D year^−1^ in the Balkans. For Ljubljana, GDDgrow changes are larger, but research has already confirmed that GDD models can vary by space, species or method. Microclimate must also be considered, as plants in urban areas may require less GDDgrow than in rural areas for the onset of a particular developmental phase (Chamberlain and Wolkovitch 2023). Temperature in the mid- latitudes where Ljubljana is located is the most important environmental factor affecting biota, both the development of plants and the life cycle dynamics of many insects. The consequences of a change in the amount of heat can significantly alter the environment in which plants grow in urban areas, such as shifts in plant phenology — the timing of recurring events in its life cycle — with consequences for their general conditions and the ecological and health services they provide (Wohlfahrt et al. 2019).

Global warming is also expected to trigger an expansion in the abundance and geographic range of some insect species and could increase the risk of insect-borne diseases. Over the last decade, the risk of locally transmitted outbreaks of vector-borne diseases accelerated by extreme heat has increased in Europe. Projections indicate a further geographical spread of various infections influenced by climate change, such as tick-borne diseases or West Nile virus outbreaks (Alkishe et al. 2017; Paz 2020). In Slovenia, we are also confronted with the above-mentioned problems (Grgič-Vitek and Klavs 2011). Therefore, it is important to study the effects of a warming climate on biota in urban environments, which can serve us as field laboratories.

The results of our study clearly show a gradual increase in frequency and severity of heat waves in Ljubljana. A similar pattern was found for the annual number of HW events in the Continental Europe biogeographical region, where the HWN increased from an average of 2.3 events/year in the period 1961–1990 to 4.1 events/year in the period 1991–2018, with an average rate of 0.5 events/decade (Oliveira et al. 2022). Several other studies have also found upward trends in HW-related indices in the European regions where heatwave frequency shows the fastest and most significant changes (Perkins-Kirkpatrick and Lewis 2020). Accordingly, the strongest HWF upward trends were found in the Mediterranean region with an HWF increase rate of 3.7 days/decade, and in the continental region the rate of change was 2.8 days/decade (Oliveira et al. 2022). Our results for warm spell duration are consistent with those of Mateus and Potito (2022), who found an increase in WSDI (+ 3.9 days) from 1885 to 2018 in Ireland, and with those of Christidis and Stott (2016), who found a positive trend between 2.3 and 2.9 days in Europe for the period 1961–2010. Exposure of Ljubljana residents to increasing heat can significantly affect the quality of life and cause health problems (Pogačar et al. 2018). Heatwave-related deaths have already increased in Slovenia recently; the most vulnerable population during heatwaves were elderly people (Perčič et al. 2018). Social status is also a very important factor, as socially disadvantaged populations, such as people with low incomes or those living in social isolation, are more exposed to the risk of heat stress and have a much lower adaptive capacity. It is therefore important to take these social inequalities into account when planning heat protection measures, interventions and adaptation strategies (Asseng et al. 2021; Slesinski et al. 2025).

Ljubljana has already undertaken the first climate mapping of the city in 2000, which drew attention to the obvious heat island in Ljubljana and emphasized the great importance of appropriate future spatial planning to reduce the impact of the urban heat island (Komac et al. 2016). Since then, great efforts have been made to make the city healthier and more sustainable. Ljubljana was awarded the prestigious title of European Green Capital in 2016, the European Best Destinations organization placed Ljubljana at the top of the selection of the twenty best green capitals in Europe in 2021, and in 2024 the Arbor Day Foundation and the Food and Agriculture Organization of the United Nations (FAO) awarded Ljubljana the title of Tree City (City of Ljubljana 2024) for the fifth time in a row as part of the Tree Cities of the World program. Despite all these measures, there are still many problems with heat stress in the city, and in some places the heat risk has recently become even greater. The proportion of built-up and traffic-loaded areas is constantly increasing; according to the city’s strategic spatial plan, the biggest changes are planned within the city ring road, where the processes of land use change will mainly go in the direction of expanding residential areas, and in some cases also retail, commercial and industrial areas (Lampič 2018). In addition, the suburban areas around Ljubljana are also increasingly becoming built-up areas (Žiberna and Konečnik Kotnik 2020). The development of the city and in particular the construction of many shopping centres with large car parks have increased the intensity of the urban heat island phenomenon. On the other hand, shopping centres could potentially provide air-conditioned spaces for certain groups of citizens who cannot afford air conditioning at home during hot days (Amorim-Maia et al. 2023).

High heat pollution in summer has numerous negative consequences, as it affects the health and mortality of the population (Perčič et al. 2018, 2024), reduces productivity, especially in demanding mental and physical work, which significantly increases the possibility of injuries in the workplace (Črepinšek et al. 2023; Pogačar et al. 2020).

A large group of organizations and individuals wrote an open letter to the Mayor of Ljubljana during the extremely hot summer of 2022, arguing that the Municipality of Ljubljana should start preparing a strategy against the heat and the consequences of climate change in the city as soon as possible and emphasizing the importance of long-term and effective measures to cool the city (City of Ljubljana 2024). When planning measures, it is important to consider that despite the relatively small size of the city of Ljubljana, there are large differences in microclimate even over short distances, based on the type of urban configuration, which are also typical for some other small cities (Iungman et al. 2024). Ljubljana has a specific configuration, a ‘star-shaped’ spatial form defined by the main transport corridors connecting the centre with the outskirts, and intersected by green edges, Golovec, Castle Hill and park Tivoli, which extend deep into the city centre. These green wedges provide ecological and climatic connections between the urban part of the city and its natural hinterland (Žlender and Gemin 2020). A study of summer temperature conditions in Ljubljana in two nearby residential neighborhoods with similar morphological structure and climatic conditions showed that one of the neighborhoods has a significantly higher UHI intensity than the other. The most important parameters for the cooling effect were green spaces and their placement as well as the absence of traffic areas, especially stationary traffic (Fikfak et al. 2017). Measurements of the surface temperature of roads and sidewalks using infrared cameras in summer also showed that the differences between shaded and unshaded areas over a very short distance were more than 20 °C (Kučić 2018).

As in many other cities (Lehnert et al. 2023; Leichtle et al. 2023), Ljubljana has recently adopted the approach of mapping hotspots based on citizens’ perception of the microclimate. The ‘Hotspots Ljubljana project’ has shown that people choose longer routes in summer or avoid certain places in the city because of the heat, some even prefer to use cars instead of cycling or walking to escape the sun, and that bus stops are also often the hotspots (Hotspots Ljubljana 2024). All this shows that although much has already been done to prevent heat stress, there are still many opportunities to improve thermal discomfort and reduce heat stress in the urban areas of Ljubljana. A better insight into the parts of the city where it gets too hot is urgently needed. As part of the heat island study in the Municipality of Ljubljana, temperature and humidity measurements were started in July 2024 at various locations in the city. Based on this data, a digital platform will provide real-time insight into the dynamics of temperatures at these locations. The aim is to create a heat island map of the city for different times of day and individual parts of the city, different seasons and weather types (City of Ljubljana 2024). These heat maps will identify hot spots in the city and show where cooling measures are most urgent. Possible measures to mitigate the summer heat include creating more green and water areas, increasing the size of urban forests in Ljubljana and promoting urban gardening (Rural development and urban farming strategy 2022). It is important to consider social inequalities when planning heat stress responses and implementing climate change adaptation measures (Slesinski et al. 2025).

Due to the city’s basin location and the resulting relatively poor ventilation, it is particularly important to preserve the existing green wedges and, if possible, to plan new ones to increase the inflow of cooler air. Appropriate urban planning is required for the future, focusing on the selection of low albedo materials for buildings, roads and sidewalks that reflect light better, retain less heat and consequently improve thermal comfort. Fewer asphalt and concrete areas, fewer parking lots and wide streets that are not blocked by tall skyscrapers are also goals that are already partly enshrined in Ljubljana’s development strategy (Sustainable Urban Strategy 2020). With further climate changes, the citizens of Ljubljana will be even more exposed to summer temperature extremes (Bertalanič et al. 2018). Cities in northern latitudes will experience the most dramatic shifts in extreme temperature conditions. Ljubljana, a city located on the border between two climate zones most affected by global warming – the Alps and the northern Mediterranean - is expected to experience the largest increase in average temperatures in the summer months, according to a forecast by the Swiss institute Crowther Lab (Bastin et al. 2019). The climate of the city of Ljubljana is expected to become a humid subtropical climate by the end of the century, like many other cities in Central Europe (Beck et al. 2018), meaning that hot and humid summers could increase the risk of heat stress. Therefore, it is of utmost importance to monitor temperature conditions and analyze their spatial and temporal changes to take appropriate adaptation and mitigation measures. The public should be informed and warned about heat events through a heat warning system to reduce the harmful effects on health. Recently, the Slovenian National Meteorological Service has made significant progress in providing a specific heat forecasting and warning system. The Universal Thermal Climate Index (UTCI) as an indicator of heat-related health risks is published on the internet three days in advance, together with an explanation of the categories related to heat stress for specific locations by hour (ARSO 2024b). The National Institute of Public Health gives detailed instructions on how to adapt behaviour in case of HW (National Institute 2025), but some additional measures are needed based on the recommendations given.

If we had had temperature data for different locations in the city, not just from the single official weather station, we might have obtained different results for the largest shopping center and the industrial areas, which would probably have an even greater impact than the one we report here. Furthermore, to assess thermal stress based on a proven increase in heat exposure in Ljubljana, morphological urban parameters as well as thermal comfort indices, which include humidity, wind and radiation in addition to temperature (Blazejczyk et al. 2012), should be evaluated. Future research could benefit from the combination of these three groups of variables – weather parameters, urban characteristics and human thermal comfort parameters. Some of the necessary data mentioned above is already available, but much more is planned for the near future.

## Conclusions

A long-term study of air temperatures for Ljubljana showed an increase in heat exposure in summer over the last 75 years. The observed increase in air temperature was greater for extreme temperatures than for average temperatures. Due to these changes, the number of hot days, tropical nights, intensity, frequency and duration of heatwaves have also increased. The cumulative temperature totals, heating degree days and cooling degree days have also changed significantly, which means that less energy has been used to heat buildings and more to cool them in recent years. Particularly pronounced changes have been observed since the 1990s. Today, air temperatures of over 35 °C in summer are no longer a rarity, which was still an extremely rare phenomenon two decades ago. Periods of hot weather and heatwaves start earlier and can last until late fall – citizens are exposed to the heat for longer, which worsens the quality of life in the city. Despite many measures already implemented to reduce heat stress, numerous problems remain and in some places heat stress is even increasing. Unfortunately, the models also show that the situation could worsen with climate change. It is necessary to continue monitoring temperature conditions with local and temporal changes and the impact of social status, and to prioritise the creation of an urban climate map, also based on building structure. All this could contribute to the implementation of the comprehensive heat warning system for better heat adaptation and mitigation measures.

## Data Availability

The datasets generated during and/or analysed during the current study are available from the corresponding author on reasonable request.
